# The Contribution of Increased Gamma Band Connectivity to Visual Non-Verbal Reasoning in Autistic Children: A MEG Study

**DOI:** 10.1371/journal.pone.0163133

**Published:** 2016-09-15

**Authors:** Natsumi Takesaki, Mitsuru Kikuchi, Yuko Yoshimura, Hirotoshi Hiraishi, Chiaki Hasegawa, Reizo Kaneda, Hideo Nakatani, Tetsuya Takahashi, Laurent Mottron, Yoshio Minabe

**Affiliations:** 1 Department of Psychiatry & Behavioral Science, Graduate School of Medical Science, Kanazawa University, Kanazawa, 920–8640, Japan; 2 Research Center for Child Mental Development, Kanazawa University, Kanazawa, 920–8640, Japan; 3 Health Administration Center, University of Fukui, Matsuokashimoaizuki, 910–1193, Japan; 4 University of Montreal Center of Excellence for Pervasive Developmental Disorders (CETEDUM), Montreal, Quebec, Canada; Chiba Daigaku, JAPAN

## Abstract

Some individuals with autism spectrum (AS) perform better on visual reasoning tasks than would be predicted by their general cognitive performance. In individuals with AS, mechanisms in the brain’s visual area that underlie visual processing play a more prominent role in visual reasoning tasks than they do in normal individuals. In addition, increased connectivity with the visual area is thought to be one of the neural bases of autistic visual cognitive abilities. However, the contribution of such brain connectivity to visual cognitive abilities is not well understood, particularly in children. In this study, we investigated how functional connectivity between the visual areas and higher-order regions, which is reflected by alpha, beta and gamma band oscillations, contributes to the performance of visual reasoning tasks in typically developing (TD) (n = 18) children and AS children (n = 18). Brain activity was measured using a custom child-sized magneto-encephalograph. Imaginary coherence analysis was used as a proxy to estimate the functional connectivity between the occipital and other areas of the brain. Stronger connectivity from the occipital area, as evidenced by higher imaginary coherence in the gamma band, was associated with higher performance in the AS children only. We observed no significant correlation between the alpha or beta bands imaginary coherence and performance in the both groups. Alpha and beta bands reflect top-down pathways, while gamma band oscillations reflect a bottom-up influence. Therefore, our results suggest that visual reasoning in AS children is at least partially based on an enhanced reliance on visual perception and increased bottom-up connectivity from the visual areas.

## Introduction

Autism spectrum (AS) manifests in early childhood as delayed, impaired, or atypical social interactions and communication, as well as a narrowing in the range of interests. AS has been frequently associated with intellectual disability, but intellectual disability may not be intrinsic to autism[1]. Autism spectrum (AS) and non-AS individuals perform differently on tasks used to estimate intelligence. AS individuals obtain better scores when evaluated with a non-verbal task, the Raven’s Progressive Matrices (RSPM) test, than with the Wechsler IQ test, a battery of tasks relying heavily on language competence. Non-AS individuals do not show this discrepancy [2–4]. RSPM is a non-verbal problem solving task which consists of finding a figure logically completing a series of complex visual patterns. Functional magnetic resonance imaging (fMRI) during RSPM solving shows an increased reliance on extrastriate cortical areas, and decreased reliance on the prefrontal cortex in AS participants relative to non-AS individuals [5, 6]. Mental rotation is another visual reasoning ability in which AS individuals are better than non-AS individuals. It consists of a same-different judgment task on three-dimensional patterns, which requires rotating objects in one's mind. AS participants manifest less activity in frontal regions relative to non-AS participants [7] during mental rotation, as they do in RSPM. Task-dependent connectivity measured during the resting state suggests over-connectivity within the visual regions, and diminished connectivity between these regions and the rest of the brain, both associated with a strong autistic phenotype [8]. These findings, which can be generalized to a large array of tasks [9], may reflect “visual thinking” [10, 11] as well as an enhanced role of perception in intelligence [5, 6].

The facility of AS individuals with visual reasoning may originate from either enhanced bottom-up, or diminished (or even optional [12]) top-down influences. Both may contribute to the increased veridical visuo-spatial perception observed in autistic adolescents or adults. Bottom-up (or feedforward) flow of information is investigated using a variety of approaches: psychophysical tasks that separately assess each dimension of low level visual input [13], fMRI that measures the activity of visual regions during tasks [5, 6], and connectivity measures [14].

Effective communication within groups of neurons along neural networks requires rhythmic synchronization or *coherence* between pre- and postsynaptic machinery [15–17]. Neuronal coherent oscillations condition the coordination and communication between neural populations that are simultaneously engaged in a cognitive process [18]. This ‘‘communication through coherence” [15] has been supported by animal studies [19–22]. For example, visual areas exert a feedforward influence on further processing through gamma band oscillation (*e*.*g*., V1 to V4) in monkeys [19, 21, 22]. A recent human magnetoencephalography (MEG) study also demonstrated that gamma band activity during a visual task using faint stimuli was positively correlated with their visual awareness [23]. Furthermore, in visual areas, feedforward projections predominately influence the gamma band [24], whereas feedback projections predominantly influence the alpha and/or beta band [24–26].

In EEG/MEG, coherence analysis measures functional connectivity through correlations between specific frequencies [27–33]. Coherence reflects the degree of phase-locking between activities recorded at different sensors which have also been used in clinical EEG and MEG [28–33]. MEG produces a reference-free signal with a very high temporal resolution, and is thus an ideal tool to compute coherence between two distant cortical rhythms. However, the magnetic fields generated by a single brain oscillator are detected by multiple sensors when the sensors are located within short distances [34]. This is usually referred to as “field spreading effects” which produce redundancies in the measurement rather than brain interaction. A way to suppress these artifacts is to employ imaginary coherence (ImCoh) analysis. ImCoh is only sensitive to synchronizations of time-lagged processes and insensitive to artefactual “spurious-interaction” [35, 36]. Therefore, in the present study, we employed ImCoh (instead of conventional coherence) to evaluate inter-sensor connectivity, which included evaluating the sensors within short distances, as well as those at long distances.

**Fig 1:**
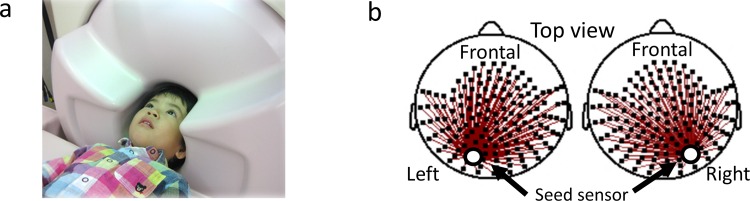
The custom child-sized MEG system and the sensor level connectivity analysis. (a) In the custom child-sized MEG system, the MEG sensors are as close to the whole head as possible for optimal recording in young children. During MEG recording, the children lay supine on the bed and viewed video programs projected onto a screen. (b) We calculated ImCoh values between the seed sensor (white circle) and the other 146 sensors (red lines).

The present study investigates cortical network connectivity in 4 – 10-year-old children during passive visual information processing using custom child-sized MEG system (Fig 1A) in which sensors were located as close to the brain as possible for optimal recording in young children [37]. Alpha, beta, and gamma band oscillation were used as proxies for bottom-up and top-down information processing to assess their contribution to visual reasoning (*e*.*g*., mental rotation and matrix analogies tasks) in AS children [24].

## Materials and Methods

### Participants

All participants were recruited from public nursery schools in Kanazawa city, Kanazawa University Hospital, and prefectural hospitals in Toyama. Children were diagnosed by a clinical psychiatrist and a clinical psychologist with more than five years of experience in AS using the Autism Diagnostic Observational Schedule–Generic (ADOS) [38], the Diagnostic Interview for Social and Communication Disorders (DISCO) [39], and the DSM-IV [40] criteria at the time the children participated in this study. AS children were included in this study when they fulfilled the diagnosis of childhood autism (66.7% of all included subjects), atypical autism (27.8%), or Asperger’s syndrome (5.6%) by the DISCO. One patient did not meet the ADOS criteria for the autism spectrum; however, we included her because she fulfilled the criteria of atypical autism by the DISCO. The exclusion criteria included known hearing loss or intellectual disability. The children’s cognitive skills were assessed by the Japanese version of the Kaufman Assessment Battery for Children (K-ABC) [41]. We excluded those subjects whose standardised score of the K-ABC (the average value of cognitive and achievement scales) was below 70 (i.e., 2 standard deviations below the mean).

Twenty-one AS children initially participated in the experiment. We excluded three children, one because their standardised score on the K-ABC scales was below 70 (i.e., intellectual disability), one because he could not follow our instructions, and one who had a reaction time over three standard deviations from the mean reaction time. We finally used data from 18 children for further analysis. The participants had a mean age of 83.9 months (range: 59–111). The mean standardised K-ABC score was 102.6 (range: 76.5–130.0). The TD group was matched to the AS group for chronological age, gender, and standardised K-ABC score (Table 1). The performances (raw score) on all Kaufman Assessment Battery (K-ABC) subtests of the children with ASD and the TD young children are shown in the S1 Fig. All TD children were native Japanese and had no prior or current developmental, learning, or behavioral problems, as was reported on a questionnaire that was completed by their parents. All the children had normal visual acuity according to their available medical records, i.e., they had never been noted to have any problems with visual acuity at three-year-old routine health checkup, and they displayed no problems with visual acuity in their daily lives. Written informed consent (by the parents) was obtained prior to enrolment in the study. The Ethics Committee of Kanazawa University Hospital approved the methods and procedures, all of which were in accordance with the Declaration of Helsinki.

**Table 1:** Demographics of the participants.

	TD	AS
Number of subjects	18	18
Gender (male/female)	13/5	13/5
Chronological age (month)	85.9 (54–122)	83.9 (59–111)
Dominant hand (right/left)	17/1	16/2
ADOS score	-	11.4 (6–17)
Standardised score of the K-ABC scales	104.9 (77.5–125.5)	102.6 (76.5–130.0)

Standardised K-ABC score; the average value of cognitive and achievement scales.

### Visual reasoning task

The scores of the Matrix Analogies subtest in the K-ABC [41] represent visual reasoning ability. The Matrix Analogies test requires the selection of a picture or design that best completes a visual scene or pattern. The examiner conveys the instructions through gestures, and the child responds with hand movements as in Raven’s Progressive Matrices test [42], a nonverbal measurement test.

### Mental rotation task

Individuals’ 3-D mental rotation ability was measured using part of the Purdue Spatial Visualization Tests: Visualization of Rotations (PSVT: R) [43–45]. We employed the first 10 questions of the mental rotation task. Participants were required to study how the object on the top line is rotated (S2A Fig). The next picture was presented at the participants’ request and they were required to picture in their mind what the object shown on the middle line (S2B Fig) looks like when rotated to match the image of the top line. They were then required to indicate the correct number from the five drawings (1, 2, 3, 4, or 5) shown on the bottom line, as quickly as possible (S2B Fig). The experiment consisted of two exercises and 10 trials with a *ca*. 3–10 second break between the pictures based on the participant’s desired timing. The period from the time the second picture was presented to the time they answered was defined as the reaction time. We calculated a value (efficiency score) to control for speed–accuracy trade-offs in the mental rotation outcome data by dividing the number of correct answers by the mean response time (RT) of all responses for each subject. For example, an average RT of 10 sec and six correct answers would yield an efficiency score value of 0.6 (/sec). A higher score indicates better performance. The visual tasks were generated using the software package SuperLab 4.0 (Cedrus, San Pedro, CA, USA). The number of correct answers and RT was measured and recorded by an examiner pressing a button.

### Magnetoencephalography recordings

MEG data were recorded using a 151-channel SQUID (Superconducting Quantum Interference Device), whole-head coaxial gradiometer MEG system for children (PQ 1151R; Yokogawa/KIT, Kanazawa, Japan) (Fig 1A) in a magnetically shielded room (Daido Steel, Nagoya, Japan). Staff members made contact with the children and played with them along with the parent(s)/caretaker(s). We confirmed the position of the head to be in the center of the MEG helmet by measuring the magnetic fields after passing currents through coils that were attached at three locations on the surface of the head as fiduciary points with respect to the landmarks (bilateral mastoid processes and nasion). MEG data were acquired using a sampling rate of 1000 Hz and filtered using a 200 Hz low-pass filter. The MEG session lasted six min. During MEG recording, one staff member (author YY) escorted each participant into the shielded room, which was decorated with colorful pictures of Japanese (cartoon) characters. During the MEG recording, the children passively viewed a video program projected onto a screen, selected according to the preference of each participant. This type of visual stimulation is an effective way to keep the young children’s attention focused on the screen and their heads still during the MEG recording. It enabled us to obtain enough artifact-free segments (80–82 sec periods in total) for connectivity analysis. None of the participants endured high emotional tension or any other kind of discomfort during the measurements based on their evaluation.

### Data analysis

#### Performance on the mental rotation task and the Matrix Analogies subtest in the TD and AS groups

The number of correct answers and reaction times between TD and AS children were compared using unpaired, two-tailed t-tests. The raw scores on the Matrix Analogies subtest in the TD and AS groups were also compared using unpaired, two-tailed t-tests. A Pearson’s correlation was used to determine significant correlations between performance on the mental rotation task (the number of correct answers/reaction time of all responses) and the raw score on the Matrix Analogies subtest of the K-ABC in the TD and AS groups. The significance level was set at P < 0.05.

#### Imaginary coherence analysis with MEG data

We employed ImCoh values between the occipital and other areas during passive exposure to a video program as a physiological index of the brain network. Offline analysis of the MEG data was performed using Brain Vision analyzer 2 (Brain Products GmbH, Gilching, Germany) software. In 151-channel SQUI, we excluded four sensors because they were malfunctioning for some subjects. We finally used 147 sensors for further analysis. The MEG data were resampled at 500 Hz. The data were split into 2 sec segments. Artifact-free segments were selected based on visual inspection. The process of eliminating contaminated data was performed blindly. Eighty sec periods were sufficiently long to obtain reproducible results for resting state power analyses [46–48]. In the present study, MEG spectra were calculated for 40 to 41 artifact-free segments (80–82 sec period) using fast Fourier transformation (FFT) with a spectral resolution of 0.5 Hz. Coherence (Cross-Spectrum/Autospectrum) is usually calculated following a Fourier transformation using the formula: Coherence (c1, c2)(f) = | CS(c1, c2)(f) |² / (| CS(c1, c1)(f) | | CS(c2, c2)(f) |), in conjunction with CS(c1, c2)(f) = Σ c1, i (f) c2, i (f)* (CS, Cross-Spectrum). In the second formula, totaling is carried out via segment number i. Calculation of the average also relates to segments with a fixed frequency, f, and a fixed channel, c. Using this methodology, values between 0 and 1 are obtained for each frequency and each channel. We employed imaginary coherence (ImCoh) analyses which were calculated using the square of the imaginary part of the cross-spectrum (imagCS) instead of the cross-spectrum for the coherence analysis. The formula is as follows: ImCoh (c1, c2)(f) = imag(CS(c1, c2)(f))² / (| CS(c1, c1)(f) | | CS(c2, c2)(f) |).

**Fig 2:**
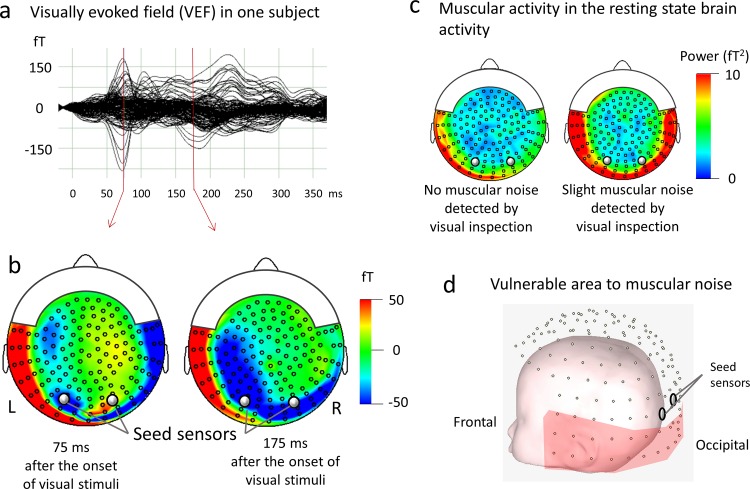
Seed sensors for high signals from visual cortices and low noise from muscular activity. (a) A representative example of the magnetic responses to the visual stimuli obtained from one subject (8-year-old boy). MEG waveforms (147 channels) are overlaid at the corrected baseline. (b) Isocontour maps of the magnetic field after pattern reversal visual stimuli. The magnetic field strength is indicated by color, varying from blue (flux-in) to red (flux-out)) at the response peak 75 and 175 msec after pattern reversal. Two seed sensors are indicated by gray circles and the other 146 sensors are indicated by dots. In two seed sensors, signals from visual cortices (i.e., visually evoked response) were optimally recorded. (c) Contamination of muscular activity in resting state brain activity. Topography of gamma band (30–58 Hz) power during rest with (right) and without (left) visually confirmed muscle activity. Sensors located in the ventral area (right) are vulnerable to muscular noise. (d) Vulnerable areas to muscular noise are close to the muscles of head and neck (e.g. temporal muscles). Two seed sensors indicated by gray circles are located outside of these vulnerable areas to muscular noise.

Artifact-free (e.g., muscle activities) segments were chosen by visual inspection. We selected two seed sensors corresponding to the right and left occipital area to minimize muscular noise contamination (Fig 2B and 2C). We calculated the ImCoh value between the seed sensor (selected for each right and left hemisphere) and the other 146 sensors (Fig 1B). We focused on the alpha (7–12 Hz), beta (13–29 Hz) and gamma (30–58 Hz) band ImCoh asa proxy for bottom-up and top-down functional connectivity between the occipital visual area and the other brain areas [24]. As shown in the S3 Fig, we determined the alpha range to be 7–12 Hz because the alpha peak frequencies that were observed in the current study were within 7–11 Hz. The frequency of the power supply for the MEG system was 60 Hz.

#### Statistical Analysis

Unpaired t tests were performed to compare the AS and TD groups for z-transformed ImCoh in the gamma band. A Pearson’s correlation was used to determine significant correlations between the z-transformed ImCoh and performance on the mental rotation task (the number of correct answers/reaction time of all responses) or the raw score on the Matrix Analogies subtest of the K-ABC. We choose a significance level of 0.00034 (= 0.05 / 146) because of the multiple comparison of 146 connections between a seed sensor and other sensors. When significance was found with this conservative level, as a complementary approach, an alpha level of 0.05 was also used, with the risk of increasing the chance of Type I error.

### Results

#### Imaginary coherence analysis with MEG data in TD and AS children

There was no significant difference in alpha band (7–12 Hz), beta band (13–29 Hz), or gamma band (30–58 Hz) ImCoh for any sensor pair between TD and AS children, using a conservative alpha level of 0.00034 (S4 Fig).

#### Performance on a mental rotation task in TD and AS children

**Fig 3:**
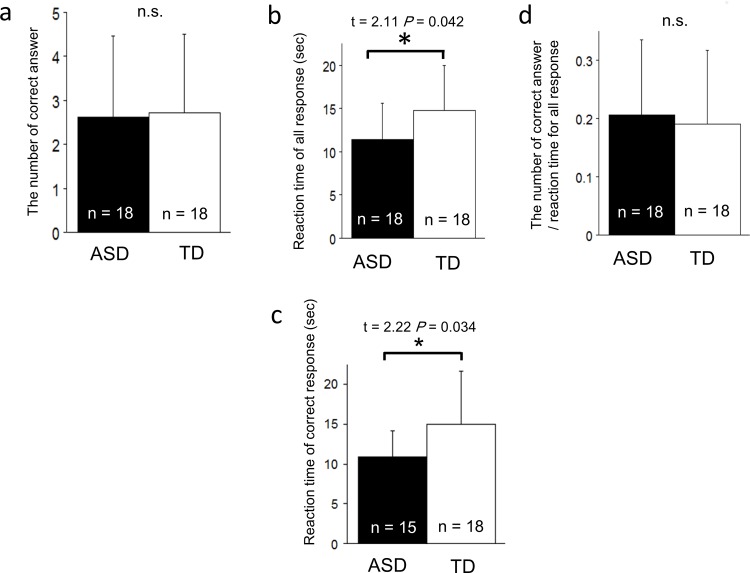
The performance of TD and AS children on a mental rotation task. No significant difference was found for the number of correct answers (a) and the efficiency index (d). AS children responded more quickly than TD children for all responses including incorrect answers (b) and for correct responses excluding incorrect answers (c). The error bars represent one standard deviation.

There was no difference in the number of correct answers between TD and AS children (t = 0.18, *P* > 0.05; Fig 3A) nor in the efficiency index (i.e., number of correct answers/reaction time) (t = 0.38, *P* > 0.05; Fig 3D), whereas the response time was shorter for AS children than TD children both for all responses (t = 2.11, *P* = 0.042; Fig 3B) and for correct responses (t = 2.22, *P* = 0.034; Fig 3C). AS children responded faster than TD children for the mental rotation task.

#### Performance on “Matrix Analogies” in TD and AS children

The mean (± SD) raw score of the “Matrix Analogies” K-ABC subtest was 8.39 (± 5.16) and 9.39 (± 4.37) for TD and AS children, respectively. There was no difference for this score between TD and AS children (t = 0.63, *P* > 0.05).

#### Correlation between performance on a mental rotation task and on a “Matrix Analogies” subtest

There was a significant correlation between performance on the mental rotation task (the number of correct answers/reaction time of all responses) and the raw score on the Matrix Analogies subtest of the K-ABC in AS children (r = 0.844, *P* < 0.001), whereas there was no significant correlation in TD children (r = 0.201, *P* > 0.05).

#### Performance on a mental rotation task and imaginary coherence in TD and AS children

**Fig 4:**
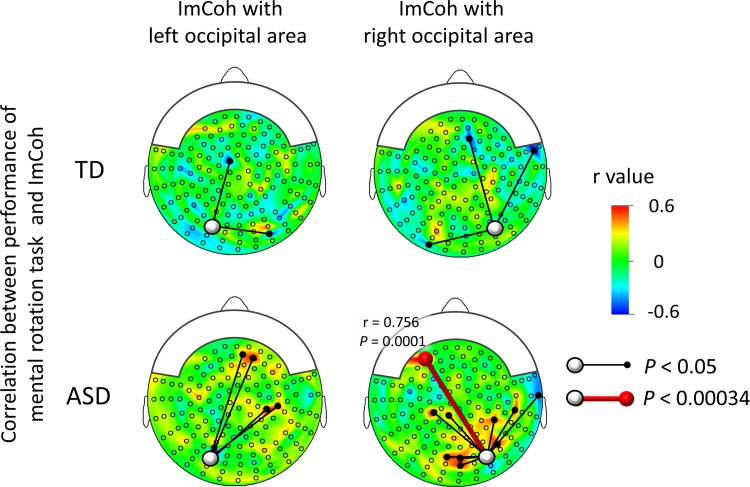
Correlation between performance of a mental rotation task and ImCoh. In TD children (upper row), there were no significant correlations for any sensor pair (i.e., alpha < 0.00034). In AS children (lower row), there was a significant positive correlation for one sensor pair (red lines); right occipital–left frontal pair (r = 0.756, P = 0.0001). ImCoh: imaginary coherence.

There was no significant correlation between task performance and ImCoh for the alpha band (7–12 Hz) or beta band (13–29 Hz) for any sensor pair either in TD or AS children, using a conservative alpha level of 0.00034. For the gamma band (30–58 Hz), there was a significant positive correlation for one sensor pair in AS children using an alpha level of 0.00034 (Fig 4, lower row; S5A Fig, r = 0.756, *P* = 0.0001), whereas there was no significant correlation in TD children. Using an alpha level of 0.05, there was a correlation between the performance of the mental rotation task and z-transformed gamma band ImCoh in AS children for 4 of 146 sensor pairs and 11 of 146 sensor pairs from the left and right occipital areas, respectively (Fig 4, lower row). The number of sensor pairs for which significant correlations were found for the ImCoh from the right occipital area exceeded the number expected by chance (146*0.05 = 7.3).

We wanted to evaluate the possible existence of a gender and/or age effect on the significant relationship (r = 0.756, *P* = 0.0001) found in the gamma band ImCoh, so as a complementary analysis, we used multiple linear regression to predict the gamma band ImCoh (i.e., dependent variable) using performance on the mental rotation task, age and gender as predictors (i.e., three independent variables). We used a significance level of *P* < 0.05. In the multiple regression model, performance on the mental rotation task was a significant predictor of the gamma band ImCoh (n = 18, β = 0.606, *P* = 0.0063), whereas age (n = 18, β = 0.241, *P* > 0.05) and gender (n = 18, β = - 0.111, *P* > 0.05) were not statistically significant predictors.

#### Performance on “Matrix Analogy” and imaginary coherence in TD and AS children

**Fig 5:**
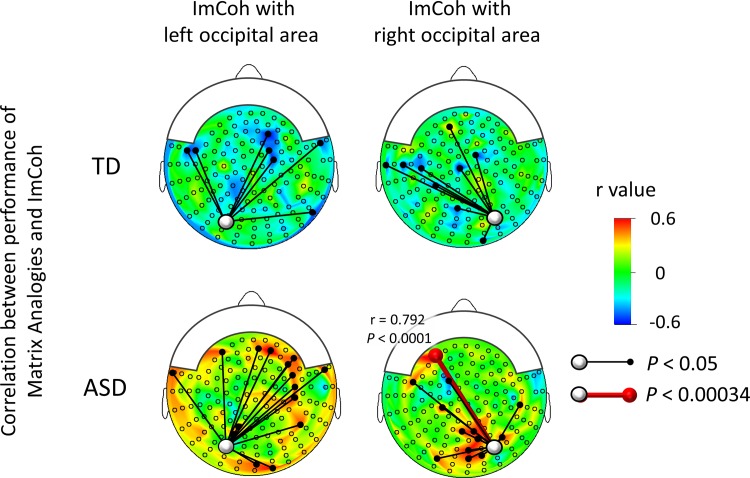
Correlation between performance on Matrix Analogies and ImCoh. In TD children (upper row), there were no significant correlations for any sensor pair (i.e., alpha < 0.00034). In AS children (lower row), there was a significant positive correlation for one sensor pair (red lines); right occipital–left frontal pair (r = 0.792, P < 0.0001). ImCoh: imaginary coherence.

There was no significant correlation between the task performance and ImCoh for the alpha band (7–12 Hz) or beta band (13–29 Hz) for any sensor pair either in TD or AS children, using a conservative alpha level of 0.00034. For the gamma band (30–58 Hz), there was a significant positive correlation for one sensor pair in AS children using an alpha level of 0.00034 (Fig 5, lower row; S5B Fig, r = 0.792, *P* < 0.0001), whereas there was no significant correlation in TD children. Using an alpha level of 0.05, there was a correlation between the performance of the mental rotation task and z-transformed gamma band ImCoh in AS children, for 15 of 146 sensor pairs and 13 of 146 sensor pairs from the left and right occipital areas, respectively (Fig 5 (lower row)). The number of sensor pairs for which significant correlations were found for the ImCoh from the left and right occipital area exceeded the number expected by chance (146*0.05 = 7.3).

To evaluate the possible existence of a gender and/or age effect on the significant relationship (r = 0.792, *P* < 0.0001) found in the gamma band ImCoh, we used multiple linear regression (i.e., same methods as shown in the results of mental rotation task). Performance on the “Matrix Analogy” task was a significant predictor of the gamma band ImCoh (n = 18, β = 0.705, *P* = 0.0038), whereas age (n = 18, β = 0.055, *P* > 0.05) and gender (n = 18, β = - 0.178, *P* > 0.05) were not statistically significant predictors.

### Discussion

We measured brain activity during passive visual processing and its relation to visuo-spatial performance and visual reasoning using MEG. Our main finding is that the gamma band frequency correlates with visual reasoning ability in AS children only, as measured by two distinct tasks. This suggests that feedforward (but not feedback) functional connectivity may differently contribute to visual reasoning ability in AS children as alpha-beta and gamma band oscillations in visual areas are proxies for top-down and bottom-up influence, respectively.

### Bottom-up connectivity and gamma band oscillation

The high temporal resolution of the MEG signal enables the investigation and quantification of various cortical rhythms [15, 49] involved in brain activity, the inhibitory or excitatory nature of the neurons involved in the task, and the feedforward or feedback directionality of the connectivity. Recent animal studies demonstrated that the gamma-band is the main neurophysiological index of bottom-up connectivity in the visual cortex [50]. Gamma-mediated inter-areal influences are predominantly bottom-up in monkeys, i.e. directed from V1 to V4 visual areas [21]. Gamma synchronization emerges in V1 supragranular layers, and influences V4 through feedforward projections. In turn, gamma synchronization emerges in V4 supragranular layers and influences further downstream areas. Conversely, the top-down influence from V4 to V1 originates from deep V4 layers, where beta band oscillation is dominant, and is thus mediated to a much lesser extent through the gamma band. Gamma band connectivity is thus thought to reflect the strength of feedforward processing [24]. Our results suggest that this element contributes to a larger extent to visual reasoning tasks in AS children, despite similar feedforward connectivity during visual processing between AS and TD children. These results are consistent with those of another recent MEG study [51], which measured feedforward functional connectivity during somatosensory processing. The authors concluded that increased long-range feedforward connectivity and reduced long-range feedback connectivity are likely characteristic of AS (but see [52]). In our previous study, we found a significantly higher coherence value in children with AS (5–7 years old) between the right occipital–right temporal connection than for TD children, using conventional coherence analysis for the gamma band [32]. In the present study, we failed to demonstrate significant differences in ImCoh for gamma bands between TD and AS children. This may result from the lower statistical power in the present study (i.e., smaller sample size), and/or from differences in the methodology (i.e., coherence and imaginary coherence). In the present study, children with AS who had higher gamma band connectivity also performed better in visual reasoning tasks. This result suggests that the magnitude of feedforward connectivity associated with visual information represents a neurophysiological index of autistic visual strengths [10, 53].

### Bottom-up connectivity and visual reasoning tasks in AS

Mental rotation is a visual reasoning task. The response time for the mental rotation task was shorter for AS children than TD children, whereas the response accuracy was similar for both groups, consistent with previous studies conducted with children [54] and adults [7, 54–57]. One previous neuroimaging study demonstrated lower activation of the prefrontal cortex during mental rotation tasks in subjects with AS [7]. This was interpreted as there being a smaller contribution of top-down influence on perceptual processing in AS subjects.

The matrix analogies task, an autistic strength, is a reliable measure of fluid intelligence and general intellectual ability. In the current study, in AS children performance on the “Matrix Analogies” K-ABC subtest was significantly correlated with performance on the mental rotation task. Interestingly, in relation to our findings about performance on a mental rotation task, a previous study demonstrated that when they were solving Raven matrices, AS subjects performed with similar accuracy but had shorter response times than non-autistic controls [6]. Children and adults with AS perform better on matrix analogies tasks (such as RSPM) than would be predicted by their Wechsler intelligence quotient (IQ) [2]. In addition, they perform better than TD children matched for age and Wechsler IQ [58]. During the RSPM test, AS participants showed relatively increased task-related activity in extra-striate areas (BA18), and decreased activity in the lateral prefrontal cortex (BA9) and medial posterior parietal cortex (BA7) [6] than TD participants. Using psychophysiological interaction analyses for fMRI data during an RSPM test, a recent study revealed greater connectivity between the left inferior occipital gyrus and areas in the left superior frontal gyrus, right superior parietal lobe, right middle occipital gyrus, and right inferior temporal gyrus, which increases as a function of reasoning complexity [5] in AS subjects relative to non-autistic participants.

Altogether, these findings suggest that a combination of enhanced feedforward and atypical top-down influences is involved in autistic visual capacity. This is in agreement with the conclusion of a meta-analysis of fMRI studies examining how autistic people process complex visual material [9]. Enhanced activity in the visual associative region is reliably reported in fMRI investigations of visual reasoning and language tasks in AS children. Multiple psychological and neurophysiological studies suggests that increased bottom-up and reduced [59, 60], or optional, top-down processing may characterize AS [12, 61], and determine some aspects of the AS cognitive profile. An increased feedforward flow of information from visual areas has not only been reported in autistic visual [59, 60, 62, 63] but also somatosensory [64] and auditory [65] processing. Previous studies also reported that the enhanced perceptual abilities in AS [12] and the atypical importance of complex visual perception in autistic intelligence [10] may be, at least partially, supported by higher feedforward connectivity from visual areas. Our results also support that higher bottom-up connectivity from the occipital area is associated with the higher visuo-spatial and nonverbal reasoning performance of children with AS.

### Limitations

There are some limitations of this study. First, the differences in measured ImCoh may relate to the different spatiotemporal properties of the video stimuli during the periods corresponding to the selected artifact-free segments. Second, we did not evaluate the degree to which the subjects focused on the TV program that they chose. This limitation may be compensated for by the fact that all participants concentrated exclusively on the TV programs, which was confirmed by a video monitor system. Third, we eliminated any contaminated MEG data, including data that were obtained during obvious head movements. However, differences in fine head movements between the children with ASD and the TD children could have confounded the study results. Fourth, a majority of the children with ASD in the current study were subjects of typical level of intelligence who were able to remain stationary during the MEG measurements; therefore, the findings may not apply to children belonging to other subtypes within the autism spectrum. Fifth, given that young children were examined in the current study, we were unable to obtain structural brain information onto which we could superimpose the coordinate system of the source-estimated MEG signals. However, child-friendly, open-type MRI devices and source-space imaginary coherence analyses with beam-forming methods [35] have become a popular technique to estimate functional connectivity based on MEG/EEG. Using these methods in future studies will enable us to investigate source level brain networks in young children while they are conscious.

### Conclusion

Stronger connectivity that originates in the occipital area and that is seen in gamma band oscillations (but not in the alpha and beta bands) was associated with higher performance in AS children. Given that alpha and beta bands reflect top-down pathways, and gamma band oscillations reflect a bottom-up influence, our results suggest that visual reasoning in AS children is, at least partially, based on an enhanced reliance on visual perception and an increased bottom-up connectivity from the visual areas.

## Supporting Information

S1 Dataset This dataset contains individual z-transformed imaginary coherence values between a seed sensor (left occipital) and 146 other sensors for the alpha, beta and gamma bands (XLSX). (XLSX)

S2 Dataset This dataset contains individual z-transformed imaginary coherence values between a seed sensor (right occipital) and 146 other sensors for the alpha, beta and gamma bands (XLSX). (XLSX)

S1 Fig The performance (raw score) on each Kaufman Assessment Battery (K-ABC) subtest. The error bars represent 1 standard deviation. An unpaired t-test revealed significantly lower performance in AS children compared to TD children in one subtest (“Face Recognition”). *P<0.05. (DOCX)
(DOCX)

S2 Fig Mental rotation tasks. We employed part of the Purdue Spatial Visualization Tests: Visualization of Rotations (PSVT: R). We modified the first 10 questions of the PSVT: R. (e.g., English explanations were replaced with arrows). The experiment consisted of 2 exercises and 10 trials with a ca. 3–10 second break between pictures based on the participant’s desired timing. The period from the time the second picture was presented to the time they answered was defined as the reaction time. (a) Participants were required to study how the object is rotated in this picture. (b) In the next picture, the participants were required to picture in their mind what the object shown on the middle line looks like when rotated to match the image of the top line, and were then required to call the correct number from the five drawings (1, 2, 3, 4, or 5) shown on the bottom line, as soon as possible. (DOCX)
(DOCX)

S3 Fig Grand averaged absolute power values from 147 channels. MEG spectra were calculated using a fast Fourier transform (FFT) with a spectral resolution of 0.5 Hz in all children to show the range of alpha rhythms in the participants. The absolute power values were averaged over the 147 sensors, and the overall value was the grand average of all the subjects (thick line). The broken line indicates one standard deviation. Note that the alpha peak frequencies in the examined frequency range were within 7–11 Hz. (DOCX)
(DOCX)

S4 Fig Z-transformed ImCoh in the left and right occipital areas. A relatively higher ImCoh was observed for the sensors near the seed sensor relative to the sensors located a long distance from the seed sensor for both the TD children and the AS children (upper and middle row). T-maps between the TD children (n = 18) and the AS children (n = 18) are in the lower row. There were no significant differences in the gamma band ImCohs for any sensor pair between the TD children and the AS children (i.e., P > 0.00034). ImCoh: imaginary coherence. (DOCX)
(DOCX)

S5 Fig Scatter plot of the performance of visual tasks and ImCoh. (a) Right occipital–left frontal pair (r = 0.756, P = 0.0001) for the mental rotation task. (b) Right occipital–left frontal pair (r = 0.792, P < 0.0001) for matrix analogies. ●: AS children (n = 18); ○: TD children (n = 18). Thick line: linear regression line for AS children. Thin line: linear regression line for TD children. ImCoh: imaginary coherence.
(DOCX)
