# Supplementary material for: The Contribution of Increased Gamma Band Connectivity to Visual Non-Verbal Reasoning in Autistic Children: A MEG Study
Source: PLoS One. 2016 Sep 15;11(9):e0163133. doi: 10.1371/journal.pone.0163133 (PMC5025179; doi:10.1371/journal.pone.0163133)

PLOS ONE: Supporting Information

Title: The contribution of increased gamma band connectivity to visual non-verbal reasoning in autistic children: a MEG study

**S1 Fig**


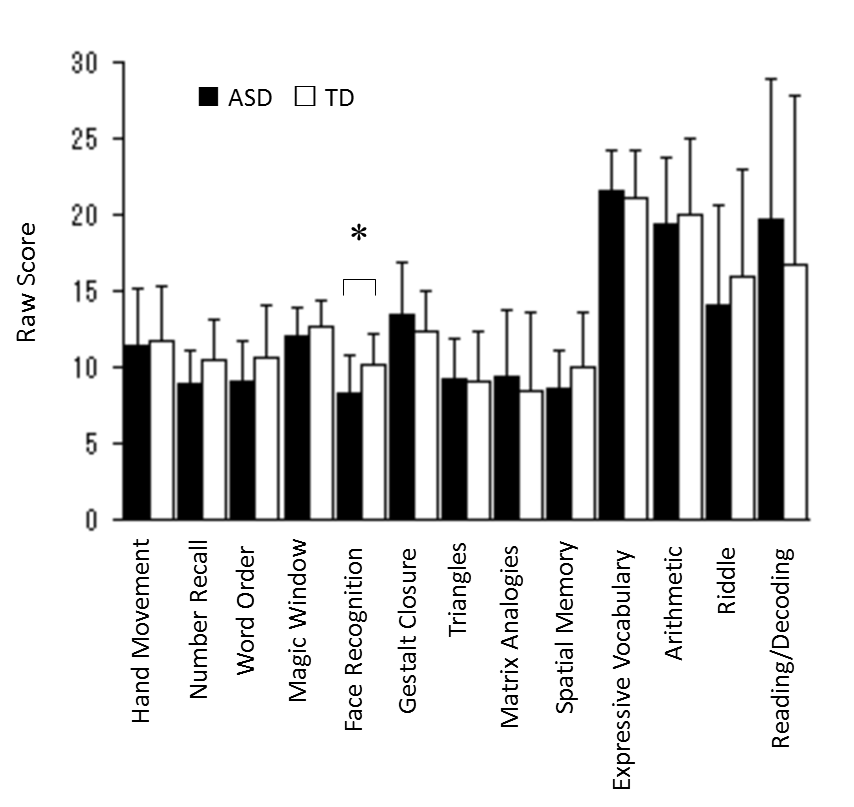

Supplement: S1 Fig — The error bars represent 1 standard deviation. An unpaired t-test revealed significantly lower performance in AS children compared to TD children in one subtest (“Face Recognition”). *P<0.05. (DOCX) (DOCX) [file pone.0163133.s003.docx]
