# Supplementary material for: The Contribution of Increased Gamma Band Connectivity to Visual Non-Verbal Reasoning in Autistic Children: A MEG Study
Source: PLoS One. 2016 Sep 15;11(9):e0163133. doi: 10.1371/journal.pone.0163133 (PMC5025179; doi:10.1371/journal.pone.0163133)

PLOS ONE: Supporting Information

Title: The contribution of increased gamma band connectivity to visual non-verbal reasoning in autistic children: a MEG study

S2 Fig


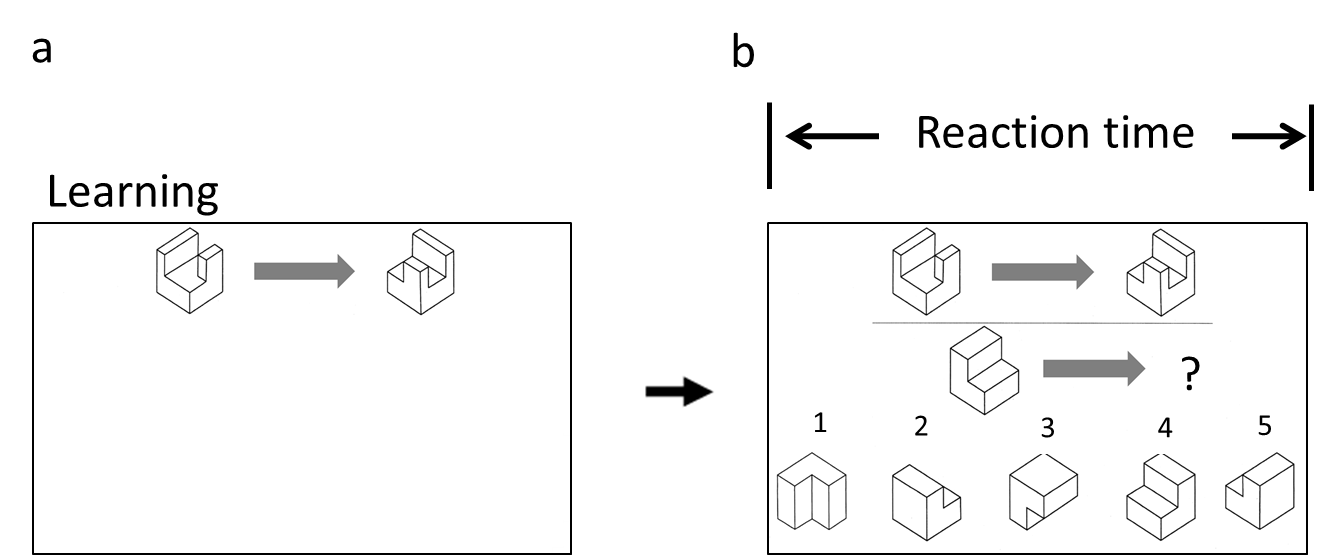

Supplement: S2 Fig — We employed part of the Purdue Spatial Visualization Tests: Visualization of Rotations (PSVT: R). We modified the first 10 questions of the PSVT: R. (e.g., English explanations were replaced with arrows). The experiment consisted of 2 exercises and 10 trials with a ca. 3–10 second break between pictures based on the participant’s desired timing. The period from the time the second picture was presented to the time they answered was defined as the reaction time. (a) Participants were required to study how the object is rotated in this picture. (b) In the next picture, the participants were required to picture in their mind what the object shown on the middle line looks like when rotated to match the image of the top line, and were then required to call the correct number from the five drawings (1, 2, 3, 4, or 5) shown on the bottom line, as soon as possible. (DOCX) (DOCX) [file pone.0163133.s004.docx]
