# Supplementary material for: The Contribution of Increased Gamma Band Connectivity to Visual Non-Verbal Reasoning in Autistic Children: A MEG Study
Source: PLoS One. 2016 Sep 15;11(9):e0163133. doi: 10.1371/journal.pone.0163133 (PMC5025179; doi:10.1371/journal.pone.0163133)

PLOS ONE: Supporting Information

Title: The contribution of increased gamma band connectivity to visual non-verbal reasoning in autistic children: a MEG study

**S3 Fig**


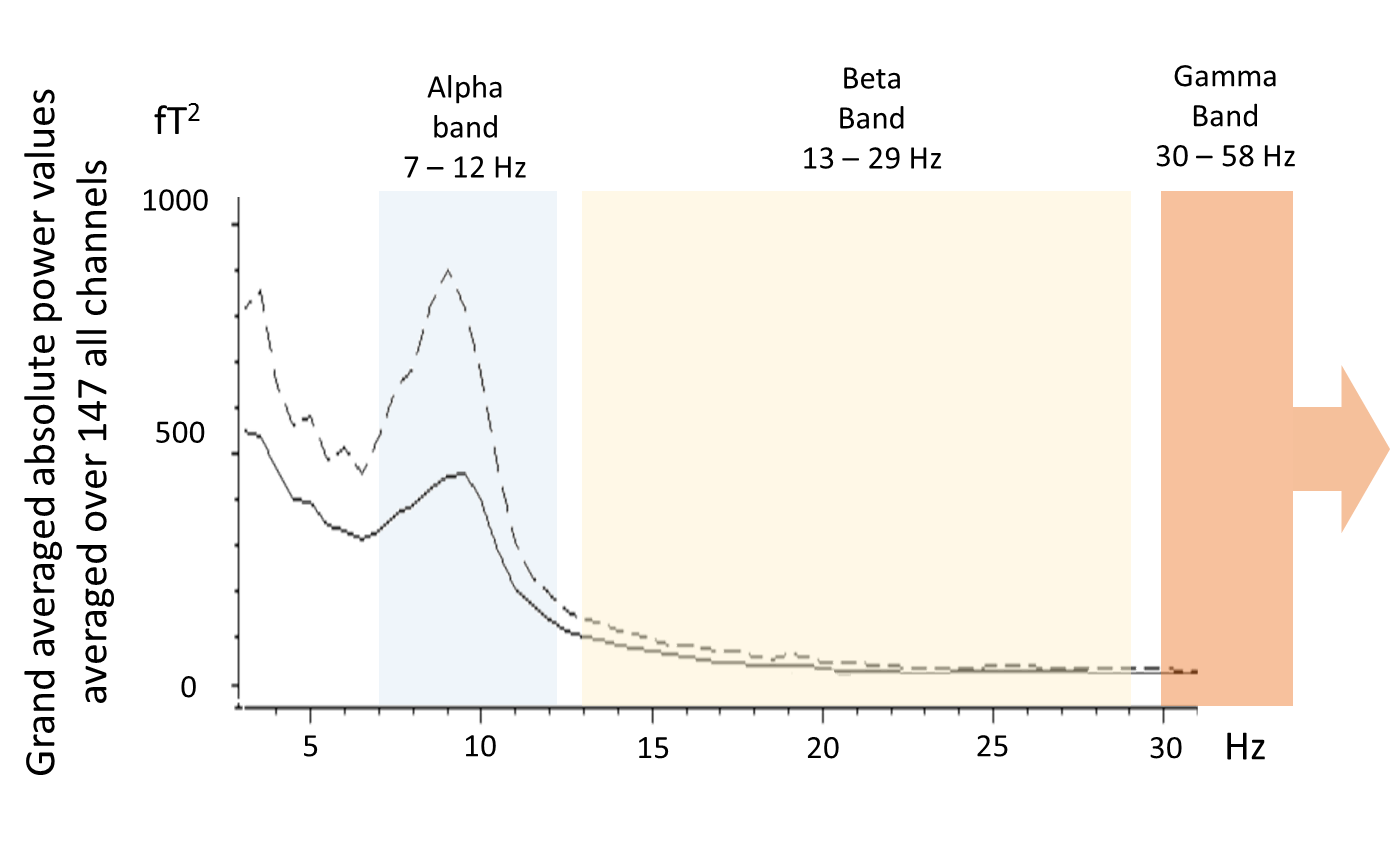

Supplement: S3 Fig — MEG spectra were calculated using a fast Fourier transform (FFT) with a spectral resolution of 0.5 Hz in all children to show the range of alpha rhythms in the participants. The absolute power values were averaged over the 147 sensors, and the overall value was the grand average of all the subjects (thick line). The broken line indicates one standard deviation. Note that the alpha peak frequencies in the examined frequency range were within 7–11 Hz. (DOCX) (DOCX) [file pone.0163133.s005.docx]
