# Supplementary material for: The Contribution of Increased Gamma Band Connectivity to Visual Non-Verbal Reasoning in Autistic Children: A MEG Study
Source: PLoS One. 2016 Sep 15;11(9):e0163133. doi: 10.1371/journal.pone.0163133 (PMC5025179; doi:10.1371/journal.pone.0163133)

PLOS ONE: Supporting Information

Title: The contribution of increased gamma band connectivity to visual non-verbal reasoning in autistic children: a MEG study

**S4 Fig**


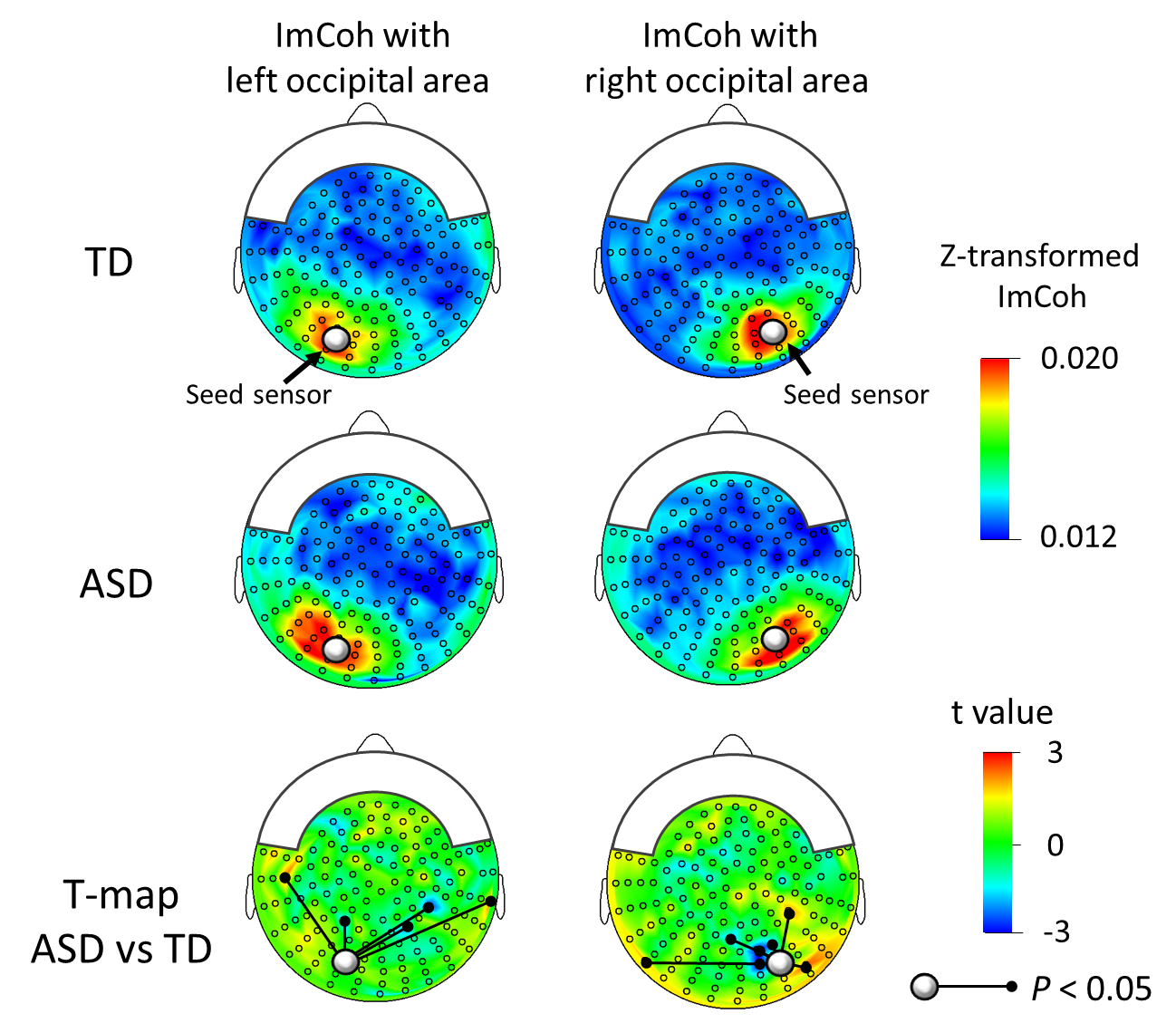

Supplement: S4 Fig — A relatively higher ImCoh was observed for the sensors near the seed sensor relative to the sensors located a long distance from the seed sensor for both the TD children and the AS children (upper and middle row). T-maps between the TD children (n = 18) and the AS children (n = 18) are in the lower row. There were no significant differences in the gamma band ImCohs for any sensor pair between the TD children and the AS children (i.e., P > 0.00034). ImCoh: imaginary coherence. (DOCX) (DOCX) [file pone.0163133.s006.docx]
