# Supplementary material for: The Contribution of Increased Gamma Band Connectivity to Visual Non-Verbal Reasoning in Autistic Children: A MEG Study
Source: PLoS One. 2016 Sep 15;11(9):e0163133. doi: 10.1371/journal.pone.0163133 (PMC5025179; doi:10.1371/journal.pone.0163133)

PLOS ONE: Supporting Information

Title: The contribution of increased gamma band connectivity to visual non-verbal reasoning in autistic children: a MEG study

**S5 Fig**


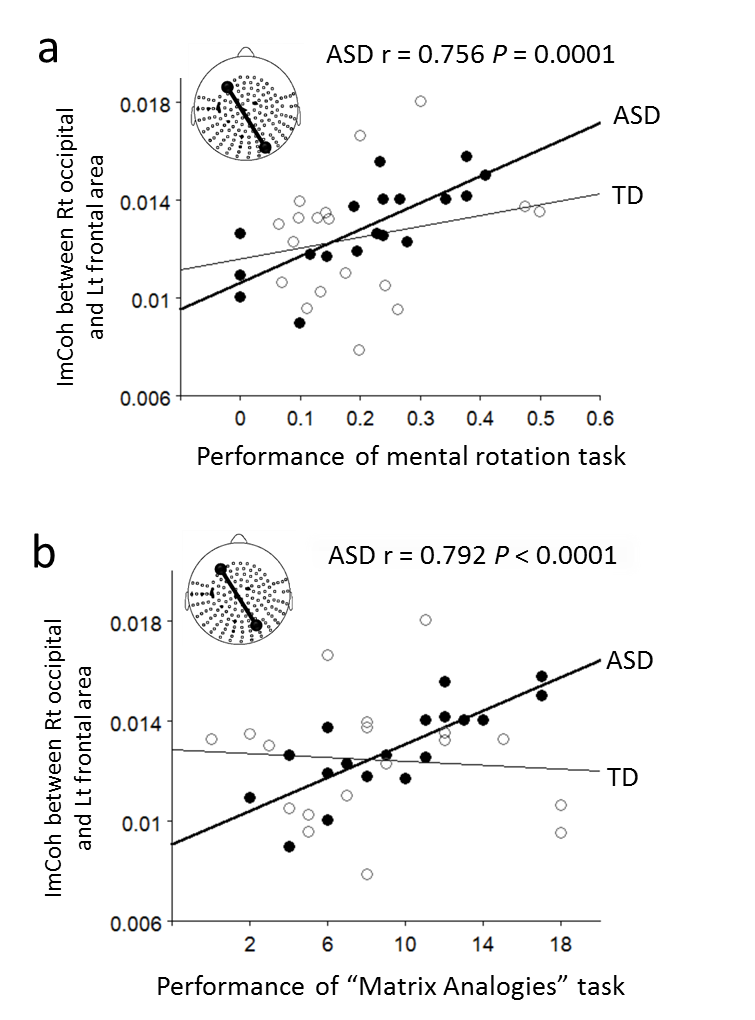

Supplement: S5 Fig — (a) Right occipital–left frontal pair (r = 0.756, P = 0.0001) for the mental rotation task. (b) Right occipital–left frontal pair (r = 0.792, P < 0.0001) for matrix analogies. ●: AS children (n = 18); ○: TD children (n = 18). Thick line: linear regression line for AS children. Thin line: linear regression line for TD children. ImCoh: imaginary coherence. (DOCX) [file pone.0163133.s007.docx]
